# Dietary Alaska Pollack Protein Induces Acute and Sustainable Skeletal Muscle Hypertrophy in Rats

**DOI:** 10.3390/nu14030547

**Published:** 2022-01-27

**Authors:** Kenji Uchida, Mina Fujitani, Takafumi Mizushige, Fuminori Kawabata, Kohsuke Hayamizu, Keisuke Uozumi, Yuma Hara, Mariko Sawai, Ryota Uehigashi, Shinji Okada, Naoko Goto-Inoue, Mizuki Morisasa, Taro Kishida

**Affiliations:** 1 The United Graduate School of Agricultural Sciences, Ehime University, 3-5-7 Tarumi, Matsuyama 790-8566, Japan; kenji_uchida@nissui.co.jp (K.U.); kishida@agr.ehime-u.ac.jp (T.K.); 2 Food Function R&D Center, Nippon Suisan Kaisha Ltd., Tokyo 105-8676, Japan; 3 Laboratory of Nutrition Science, Division of Applied Bioscience, Graduate School of Agriculture, Ehime University, 3-5-7 Tarumi, Matsuyama 790-8566, Japan; u_72keisuke@yahoo.co.jp (K.U.); yumaaa802@gmail.com (Y.H.); m-sawai@itoen.co.jp (M.S.); ryota_uehigashi@mizkan.co.jp (R.U.); 4 Department of Applied Biological Chemistry, School of Agriculture, Utsunomiya University, 350 Minemachi, Utsunomiya 321-8505, Japan; mizushige@cc.utsunomiya-u.ac.jp; 5 Physiology of Domestic Animals, Faculty of Agriculture and Life Science, Hirosaki University, 3 Bunkyo-cho, Hirosaki 036-8561, Japan; kawabata@hirosaki-u.ac.jp; 6 Laboratory of Food Chemistry, Yokohama University of Pharmacy, Yokohama 245-0066, Japan; k.hayamizu@hamayaku.ac.jp; 7 Food Functionality Laboratory, Graduate School of Agricultural and Life Sciences, The University of Tokyo, 1-1-1 Yayoi, Bunkyo-ku, Tokyo 113-8657, Japan; asoka@g.ecc.u-tokyo.ac.jp; 8 Department of Marine Science and Resources, College of Bioresource Sciences, Nihon University, Fujisawa 252-0880, Japan; naoko.goto.inoue@gmail.com (N.G.-I.); xx1mizu9xx@gmail.com (M.M.)

**Keywords:** fish protein, skeletal muscle, muscle fiber type, protein source, rat

## Abstract

Our previous studies suggested that Alaska pollack protein (APP) intake increases skeletal muscle mass and that it may cause a slow-to-fast shift in muscle fiber type in rats fed a high-fat diet after 56 days of feeding. In this study, we explored whether dietary APP induces acute and sustainable skeletal muscle hypertrophy in rats fed a normal-fat diet. Male 5-week-old Sprague–Dawley rats were divided into four groups and fed a purified ingredient-based high-fat diet or a purified ingredient-based normal-fat diet with casein or APP, containing the same amount of crude protein. Dietary APP significantly increased gastrocnemius muscle mass (105~110%) after 2, 7 days of feeding, regardless of dietary fat content. Rats were separated into two groups and fed a normal-fat diet with casein or APP. Dietary APP significantly increased gastrocnemius muscle mass (110%) after 56 days of feeding. Dietary APP significantly increased the cross-sectional area of the gastrocnemius skeletal muscle and collagen-rich connective tissue after 7 days of feeding. It decreased the gene expression of *Mstn* /Myostatin, *Trim63*/MuRF1, and *Fbxo32*/atrogin-1, but not other gene expression, such as serum IGF-1 after 7 days of feeding. No differences were observed between casein and APP groups with respect to the percentage of Type I, Type IIA, and Type IIX or IIB fibers, as determined by myosin ATPase staining after 7 days of feeding. In the similar experiment, the puromycin-labeled peptides were not different between dietary casein and APP after 2 days of feeding. These results demonstrate that APP induces acute and sustainable skeletal muscle hypertrophy in rats, regardless of dietary fat content. Dietary APP, as a daily protein source, may be an approach for maintaining or increasing muscle mass.

## 1. Introduction

The skeletal muscle is the most abundant tissue in the human body, accounting for 30–40% of adult body mass. It performs a wide variety of physiological functions; thus, maintaining skeletal muscle mass throughout the lifespan is critical for the preservation of not only independent locomotion but also metabolic health [1,2]. Any increase in mechanical loading induces skeletal muscle hypertrophy; unloading induces atrophy [3]. Therefore, resistance exercise is the preferred stimulus for maintaining muscle mass. However, improving not only mechanical loading but also food components can be beneficial for skeletal muscle mass [4,5,6,7,8,9].

Fish protein is consumed worldwide. Alaska pollack (*Theragra chalcogramma*) is utilized in processed seafoods such as imitation crab, kamaboko (fish cakes), and fish sausage. Additionally, its fillets are a popular item for fish and chips and fish sandwiches. In some parts of Japan and South Korea, dried Alaska pollack is used as a hot pot ingredient. Given the widespread use of fish protein, elucidation of the nutritional characteristics of Alaska pollack is important. In our previous studies, dietary APP increased gastrocnemius and extensor digitorum longus (EDL) muscle mass in rats fed a high-fat diet after 56 days of feeding, compared with casein [10,11]. Casein was chosen as the source of protein for the AIN-93G diet because its amino acid composition is reasonably adequate; the AIN-93G diet, and also the previous version, the AIN-76 diet, is formulated for the growth of experimental rodents as dietary standards for nutritional studies by the American Institute of Nutrition [12,13]. Therefore, we used the AIN-93G diets based on casein as control. After immobilization, dietary APP intake enhanced skeletal muscle mass, not only in immobilized limbs but also in non-immobilized limbs after 21 days of feeding in rats fed a high-fat diet [14]. Certain food components have been reported to prevent obesity-related skeletal muscle atrophy [8,9]. On the other hand, a high-fat diet has been reported to cause activation of the ubiquitin proteasome pathway that causes muscular atrophy [15]. It is important to determine whether high-fat diet feeding is essential to observe the effect of APP; however, the beneficial effects of dietary APP on the skeletal muscle of rats fed a normal-fat diet has not been addressed. Another question regards the time taken for stimulation of skeletal muscle hypertrophy by dietary APP. The skeletal muscle is a dynamic organ; it exhibits a remarkable plasticity to adapt and remodel. Inoue et al. demonstrated that the gastrocnemius muscle mass significantly increased following 3 days of electrical stimulation when compared to the gastrocnemius muscle mass in the contralateral control legs [16]. Isfort et al. demonstrated that atrophy of the soleus muscle was induced by hindlimb suspension, while hypertrophy of the soleus was induced following 2 days of reweighting [17]. In this study, we investigated the minimum duration of dietary APP to induce skeletal muscle hypertrophy.

Balance between protein synthesis and degradation maintains the skeletal muscle; if muscle synthesis exceeds degradation, the muscles become hypertrophic; if muscle synthesis falls below muscle degradation, it degrades. Autocrine/paracrine effects of local insulin-like growth factor 1 (IGF1) may be a major mechanism controlling tissue growth; protein synthesis and degradation are primarily controlled by the IGF1/protein kinase B (Akt) signaling pathway [18]. The IGF1/Akt pathway promotes protein synthesis via the mammalian target of rapamycin (mTOR) and inhibits protein degradation through the suppression of transcription of the ubiquitin ligase F-box protein 32 (*Fbxo32*; also known as atrophy gene-1 (atrogin-1)) and tripartite motif-containing 63 (*Trim63*; also known as muscle-specific RING finger protein 1 (MuRF1)) [18]. In most cases, the ubiquitin proteasome system is responsible for protein degradation [19]. Myostatin (*Mstn*) acts as a negative regulator of muscle mass that inhibits Akt activation in the skeletal muscle [18]. Muscle and solute carrier family 2 member 4 (*Slc2a4*; also known Glut4) is the principal glucose transporter protein in muscle [20]. In addition, IGF1 promotes muscle fiber regeneration and hypertrophy and was shown to increase mRNA expression of myogenic regulatory factors (MRFs) [21,22]. Substantial evidence suggests that MRFs are involved in the satellite cell responses (i.e., differentiation and fusion) of adult muscle fibers in rats [23,24,25,26,27]. In addition, hypertrophy of intermuscular tissue also occurs with muscle hypertrophy, adapted to exercise [28,29]. Our previous study showed that dietary APP significantly increased the gene expression of *Igf1*, *Myog* and *Slc2a4* [11,14].

Skeletal muscle is comprised of heterogeneous muscle fibers that differ in their physiological and metabolic parameters [30]. On the basis of the expression of specific myosin heavy chain (MHC) isoforms, muscle fibers are classified into type I, type IIa, type IId/x, and type IIb fibers, where types I and IIa exhibit oxidative metabolism and types IIx and IIb are primarily glycolytic [30,31,32]. Muscle fibers are capable of changing their phenotypic properties in response to environmental demands, exhibiting modifications in the expression of MHC isoforms. Increased neuromuscular activity, mechanical loading, and hypothyroidism are conditions that induce fast-to-slow shift occurs [31]. Our previous study showed that dietary APP increased the skeletal muscle mass, especially in fast-twitch muscle (gastrocnemius and EDL), decreased the mRNA expression of *Myh7* (gene encoding MHC I) in the soleus muscle and increased the mRNA expression of *Myh4* (gene encoding MHC IIB) in the EDL muscle [11], suggesting that dietary APP increases fast-type muscle mass and that it may cause a slow-to-fast shift in muscle fiber type. However, our previous study had limitations in terms of claiming that the muscle fiber type can be shifted from slow to fast through fish protein intake because we did not verify the change in fast-type markers at the protein level using myosin ATPase staining.

Therefore, we first examined whether dietary APP induced skeletal muscle hypertrophy after 2 or 7 days of feeding, regardless of dietary fat content, and whether dietary APP intake for 56 days maintained the hypertrophic effect in rats that were fed the normal fat diet. Second, we examined whether dietary APP skeletal muscle increased the skeletal muscle cross-sectional area (CSA), the intramuscular connective tissue, myosin ATPase activity, gene expression of MHCs, and regulators of muscle mass and metabolism after 7 days of feeding in rats fed the normal diets. Third, we explored whether dietary APP promotes protein synthesis in rats that were fed a normal-fat diet.

## 2. Materials and Methods

### 2.1. Protein Sources

Alaska pollack fillets (Nippon Suisan Kaisha, Ltd., Tokyo, Japan) were washed, freeze-dried and ground. Casein (New Zealand Dairy Board, Wellington, New Zealand) was purchased. The crude protein and crude fat contents and the composition of amino acids of each protein source are shown in Table 1.

### 2.2. Animals and Experimental Design

This study was conducted by March 2019 in accordance with the ethical guidelines of the Ehime University Animal Experimentation Committee and in complete compliance with the National Institutes of Health: Guide for the Care and Use of Laboratory Animals. All efforts were made toward minimizing the number of animals used and limiting experimentation to what was necessary to produce reliable scientific information. All protocols for the animal experiments were approved by the Ehime University Animal Experimentation Committee (permit number 08A92 (2013–2018)).

Male 5-week-old Sprague-Dawley rats (SLC, Shizuoka, Japan) were used. The rats were housed in individual stainless wire mesh cages in a room under a 12 h light–dark cycle (dark phase: 15:00–3:00) at a constant temperature (22 ± 1 °C). The animals were housed separately for acclimatization to the environment. The rats were fed regular tap water and a normal-fat AIN-93G based on casein (Cas) diet [12] ad libitum during acclimatization. The body mass and amount of food consumed were recorded every morning for each animal; the food was replenished during the acclimatization and experimental periods. After acclimatization, the rats were fed the Cas diet (high-fat or normal-fat) or the same diet in which the protein source was fully replaced by APP, containing the same amount of crude protein. Experimental diets mixed with each components of Table 2. Following the experimental period, rats were anesthetized with isoflurane and decapitated corresponding to a non-fasting state (2 h after the start of the dark phase). Blood samples were collected from the neck. The tissues and serum were stored at −80 °C and −50 °C, respectively, until analysis. The details are as follows.

Experiment 1: To confirm the effect of fat on the muscle hypertrophy by dietary APP, rats were each divided into eight groups composed of 68 rats with similar mean body mass (high-fat diet feeding for 2 days: Cas group, *n* = 8; APP group, *n* = 8; normal-fat diet feeding for 2 days: Cas group, *n* = 8; APP group, *n* = 8; high-fat diet feeding for 7 days: Cas group, *n* = 9; APP group, *n* = 9; normal-fat diet feeding for 7 days: Cas group, *n* = 9; APP group, *n* = 9, respectively). After 2 or 7 days of feeding, the skeletal muscles of the soleus, gastrocnemius, and extensor digitorum longus (EDL) were removed and weighed. The gastrocnemius muscles of the rats fed the normal-fat diet in 2-days feeding group were immediately frozen in liquid nitrogen and stored at −80 °C until gene expression analysis.

Experiment 2: To confirm the muscle hypertrophy effect of APP on normal-fat diet at long term, rats were each divided into four groups composed of 40 rats with similar mean body mass (normal-fat diet feeding for 7 days: Cas group, *n* = 10; APP group, *n* = 10; normal-fat diet feeding for 56 days: Cas group, *n* = 10; APP group, *n* = 10, respectively). After 7 or 56 days of feeding, the gastrocnemius muscles of rats in the Cas and APP groups were removed and weighed. The gastrocnemius muscles of rats in 56-days feeding group were frozen in liquid nitrogen and stored at −80 °C until analysis.

Experiments 3 and 4: To confirm an effect of the muscle gene expression and fiber composition by dietary APP on normal-fat diet, after 7 days of feeding, the gastrocnemius muscles of rats in the Cas and APP groups were removed and weighed (gene expression analysis in Experiment 3: Cas group, *n* = 10; PP group, *n* = 10; HE staining: Cas group, *n* = 7; APP group, *n* = 7; Azan staining: Cas group, *n* = 8; APP group, *n* = 8; ATPase staining in Experiment 4: Cas group, *n* = 3; APP group, *n* = 4, respectively). The gastrocnemius muscles were frozen in liquid nitrogen and stored at −80 °C until analysis.

Experiment 5: To confirm an effect of the IGF-1 by dietary APP on a normal-fat diet, After 7 days of feeding, rats in the Cas and APP groups were decapitated (Cas group, *n* = 12; APP group, *n* = 12). The blood was centrifuged at 1500× *g* at 4 °C for 10 min. The liver was removed, weighed, and frozen in liquid nitrogen.

Experiment 6: The rates of protein synthesis were measured in vivo using the SUnSET method [33]. After 2 days of feeding, rats in the Cas and APP groups were injected intraperitoneally with puromycin (4 µmol/100 g body mass in 360 µL of phosphate-buffered saline) 90 min after the start of the dark phase ((*n* = 5, Cas group and *n* = 5, APP group). Exactly 30 min after the puromycin injection, the rats were decapitated. The gastrocnemius muscle was immediately frozen in liquid nitrogen.

### 2.3. Hematoxylin and Eosin (HE) Staining

The gastrocnemius muscles were removed and immediately frozen in isopentane cooled in dry ice. Transverse sections with a thickness of 10 µm were obtained using a cryostat from the middle part of the gastrocnemius muscle and mounted on glass slides. Sequentially, the sections were incubated in Hematoxylin3G solution (Sakura Finetek Japan, Tokyo, Japan) for 10 min, rinsed with Milli-Q water for 5 min, incubated in eosin (Sakura Finetek Japan) for 10 min, and rinsed with Milli-Q water for 10 min. They were dipped in 90% ethanol for 30 s, twice in 95% ethanol for 1 min, three times in 100% ethanol for 3 min, and three times in 100% xylene for 4 min. The sections were finally mounted in a small drop of glycerol under a cover slip and observed on an inverted microscope (Olympus, Tokyo, Japan) connected to a CCD camera (Shimadzu, Kyoto, Japan). Within each image, the number of fibers and the CSA were measured semi-automatically on HE-stained sections with the aid of an imaging application (A-zoh-kun, Asahi Kasei Engineering Corp., Kawasaki, Japan). The distribution of CSA and mean CSA were determined using three images (more than 350 fibers) per gastrocnemius muscle.

### 2.4. Azan Staining

The sections were dipped for 15 min in 10% potassium dichromate solution, incubated in 0.1% azocarmine G for 30 min and subsequently rinsed with Milli-Q water 3 times. The sections were dipped in aniline alcohol (0.1 mL aniline dissolved in 100 mL 90% ethanol) for 1 min and in acetic alcohol for 1 min, rinsed with Milli-Q water 3 times. The sections were incubated in 5% phosphotungstic acid for 60 min, rinsed with Milli-Q water 3 times, incubated in aniline blue/orange G solution (0.25% aniline blue, 1% orange G, and 4% glacial acetic acid) for 60 min. They were briefly incubated in ethanol three times in 100% xylene for 4 min. The sections were finally mounted in a small drop of glycerol under a cover slip and mounted on an inverted microscope (Olympus, Tokyo, Japan) connected to a CCD camera (Shimadzu). The collagen-rich connective tissue was quantified using an imaging application (A-zoh-kun).

### 2.5. Serum IGF-1 Concentration Measurement

IGF-1 concentrations in the serum samples were measured using commercial ELISA kits (Biosensis, Pty, Ltd., Thebarton, South Australia) following the manufacturer’s instructions. Serum samples were diluted with the sample diluent buffer at 1:500. Absorbance was read at 450 nm using a spectrophotometer (UC-1200; Shimadzu, Kyoto, Japan).

### 2.6. Measuring Protein Synthesis with SUnSET

The rates of protein synthesis were measured in vivo using the SUnSET method [33]. The rats were injected intraperitoneally with puromycin (4 µmol/100 g body mass in 360 µL of phosphate-buffered saline) 90 min after the start of the dark phase. Exactly 30 min after the puromycin injection, the gastrocnemius muscle was sampled. The frozen muscles were homogenized using a Polytron homogenizer in RIPA lysis buffer (50 mM Tris-HCl, pH 7.4, 150 mM NaCl, 0.25% deoxycholic acid, 1% NP-40, and 1 mM EDTA; Merck Millipore, Billerica, MA, USA) containing a protease and phosphatase inhibitor cocktail (Thermo Fisher Scientific, Waltham, MA, USA) and centrifuged at 16,000× *g* for 40 min at 4 °C. The lysates were solubilized in Laemmli sample buffer containing mercaptoethanol and boiled. The samples (10 μg of protein) were separated on a 12% polyacrylamide gel. Proteins were then transferred to polyvinylidene difluoride membranes (Global Life Sciences Technologies Japan, Tokyo, Japan) at 100 V for 1 h. The membranes were blocked for 1 h at 18–28 °C in Tris-buffered saline (TBS) with 0.1% Tween 20 (TBST) containing 5% nonfat dry milk and incubated overnight at 4 °C with an anti-puromycin antibody (CosmoBio Co., Tokyo, Japan) or anti- COX IV antibody (3E11, Cell Signaling Technology, Danvers, MA, USA) (loading control). The membranes were washed with TBST, incubated for 1 h at room temperature with anti-mouse immunoglobulin G antibody or anti-rabbit immunoglobulin G antibody (Global Life Sciences Technologies Japan, Tokyo, Japan), and treated with an enhanced chemiluminescence reagent (ECL prime; Global Life Sciences Technologies Japan, Tokyo, Japan), following which the antibody-bound polypeptide was visualized on a ChemiDoc MP Imaging System (Bio-Rad). Densitometric measurements were performed using the ImageJ software (National Institute for Health, Bethesda, ML, USA).

### 2.7. Fiber Typing by ATPase Staining

To identify the different fiber types, myofibrillar actomyosin ATPase activity was histochemically determined using acidic pH 4.7 to identify type I fibers, as described by Lind and Kernell [34]. Transverse sections with a thickness of 10 µm were pre-incubated at room temperature for 15 min in a buffer consisting of 100 mM potassium chloride in 100 mM sodium acetate adjusted to pH 4.7 with acetic acid. Thereafter, the sections were incubated at 37 °C in 100 mM glycine–sodium hydroxide buffer (pH 9.6) containing 3 mM ATP disodium salt for 45 min. Type I dark fibers, lightly stained type IIA, and intermediate fibers of type IIX or IIB fibers were counted using an imaging application (A-zoh-kun).

### 2.8. Gene Expression Analysis

As previously described [35], the frozen gastrocnemius muscles were homogenized for isolating total RNA using Sepasol-RNA I Super G (Nacalai Tesque Inc., Kyoto, Japan) following the manufacturer’s instructions and treated with RNase-Free DNase (Takara Bio, Shiga, Japan) for 30 min at 37 °C. mRNA was isolated from total RNA using Oligotex-dT30 (Takara Bio, Shiga, Japan). cDNA was synthesized from mRNA using reverse transcriptase (Reverse Transcriptase XL (AMV) for RT-PCR, 5 U/μL, Takara Bio, Kusatsu, Japan) and Oligo (dt) primers on a thermal cycler (ABI GeneAmp2400, PerkinElmer, Waltham, MA, USA). After cDNA synthesis, qPCR was performed separately for target genes and the endogenous reference gene *Ppia*, and amplifications were performed using the StepOnePlus real-time PCR system (Applied Biosystems, Foster, CA, USA) and the THUNDERBIRD SYBR qPCR mix (TOYOBO, Osaka, Japan). The basic amplification program was set to perform 50 cycles of denaturation for 15 s at 95 °C and annealing and extension for 1 min at 60 °C. Fluorescence was recorded at 530 nm during extension. We determined the relative mRNA expression by calculating the quantification cycle (Cq) of each target gene with respect to that of the *Ppia* gene as the reference and the corresponding real-time PCR efficiency of the respective primer sets using a previously reported method [35]. The average, standard deviation and coefficients of variation of Cq (CqCV%; SD/mean·100) for *Ppia* gene for each of the different groups are provided in Supplemental Table S1. Results suggested that *Ppia* had low CqCV% (<3%), meaning that *Ppia* had a low dispersion around the mean value. Therefore, we used *Ppia* as reference gene. Primer sequences are provided in Table 3.

### 2.9. Statistical Analysis

Data are expressed as the mean ± SEM and confirmed a normal distribution by the Shapiro–Wilk test. The comparison was analyzed using three-way analysis of variance (ANOVA) with dietary protein source, dietary fat content and feeding period as factors for the data presented in experiment 1, and two-way ANOVA with protein source and feeding period as factors for the data presented in experiment 2. Multiple comparisons were assessed by Bonferroni post-hoc test. Statistical analysis was performed using Student’s unpaired *t*-test for other data. All statistical tests were performed using the IBM SPSS Statistics software (SPSS Japan Inc., an IBM company). Statistical significance was defined as *p* < 0.05.

## 3. Results

### 3.1. Effects of APP on Body Mass and Food Intake

**Figure 1:**
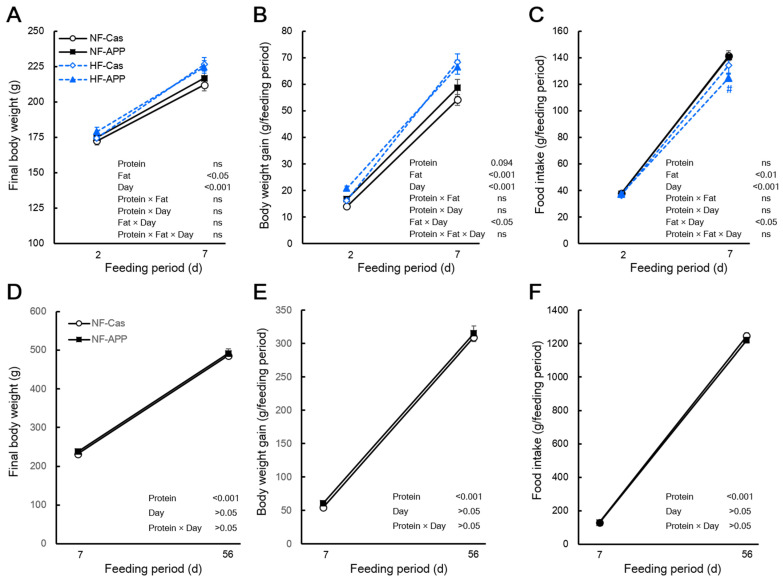
Effects of APP on the final body mass, body mass gain and food intake. The final body mass (**A**), body mass gain (**B**), and food intake (**C**) of normal-fat-casein (NF-Cas) group, normal-fat-APP (NF-APP) group, high-fat-Cas (HF-Cas) group, and high-fat-APP (HF-APP) group after 2 days of feeding (*n* = 8/group) and 7 days feeding (*n* = 9/group) in experiment 1, and the final body mass (**D**), body mass gain (**E**), and food intake (**F**) NF-Cas group and NF-APP group after 7 days of feeding (*n* = 10/group) and 56 days feeding (*n* = 10/group) in experiment 2, are shown. Data are expressed as the mean ± standard error (SEM). Statistical analyses were performed using three-way ANOVA with Bonferroni post hoc significance testing in experiment 1, and two-way ANOVA with Bonferroni post hoc significance testing in experiment 2. Asterisks indicate significant differences compared to NF-Cas group, and number signs indicate significant differences compared to HF-Cas group (^#^ *p* < 0.05). Protein, effect of protein source; Fat, effect of fat content; Days, effect of feeding period; Protein × Fat, interaction between protein source and fat content; Prorein × Days, interaction between protein source and feeding period; Fat × Day, interaction between fat content and feeding period; Protein × Fat × Day, interaction between protein source, fat content and feeding period; ns, not significant.

The final body mass of the APP group did not differ from that of the Cas group after 2 or 7 days of feeding, regardless of dietary fat content (Figure 1A). APP intake for 56 days had a similar effect under the normal-fat condition (Figure 1D). Although the body mass gain of the APP group did not differ from that of the Cas group during 2 or 7 days of feeding under the high-fat diet or normal-fat conditions, a three-way ANOVA indicated a trend toward a main effect of protein source on the body mass gain (Figure 1B). Under the normal-fat condition, APP intake for 56 days did not affect the body mass gain (Figure 1F). The food intake of the APP group did not differ from that of the Cas group during 2 or 7 days of feeding in rats fed the normal-fat diet (Figure 1C). Under the high-fat condition, the food intake of the APP group did not differ from that of the Cas group during 2 days of feeding, whereas the food intake of the APP group was significantly lower than that of the Cas group during 7 days of feeding (98.1%) (Figure 1C). However, the interaction between protein source, fat content and feeding period were not significant (Figure 1C). APP intake for 7 or 56 days did not affect the food intake under the normal-fat condition (Figure 1G).

### 3.2. Effects of APP on the Skeletal Muscle Mass

**Figure 2:**
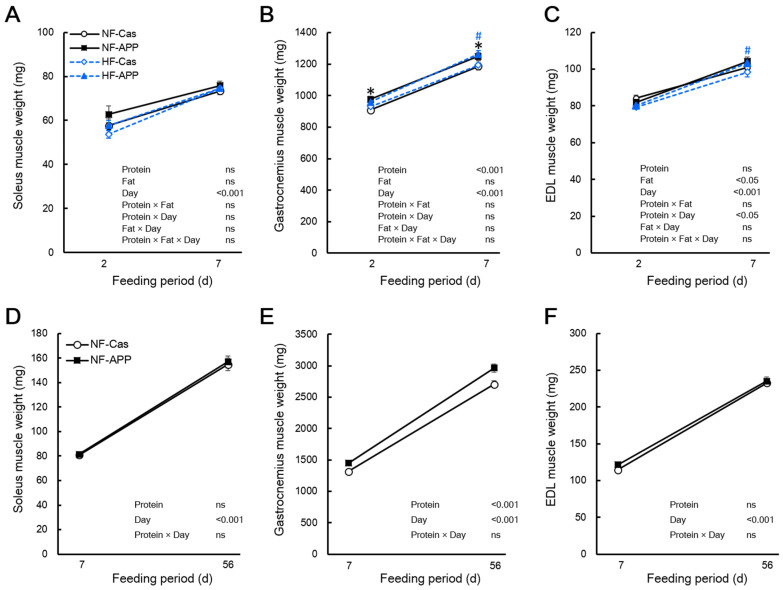
Effects of APP on the skeletal muscle mass. The soleus (**A**), gastrocnemius (**B**), and extensor digitorum longus (EDL) (**C**) of the normal-fat-casein (NF-Cas) group, the normal-fat-APP (NF-APP) group, the high-fat-Cas (HF-Cas) group, and the high-fat-APP (HF-APP) group after 2 days of feeding (*n* = 8/group) and 7 days feeding (*n* = 9/group) in experiment 1, and the soleus (**D**), gastrocnemius (**E**), and extensor digitorum longus (EDL) (**F**) of the NF-Cas group and NF-APP group after 7 days of feeding (*n* = 10/group) and 56 days feeding (*n* = 10/group) in experiment 2 are shown. Data are expressed as the mean ± standard error (SEM). Statistical analyses were performed using three-way ANOVA with Bonferroni post hoc significance testing in experiment 1, and two-way ANOVA with Bonferroni post hoc significance testing in experiment 2. Asterisks indicate significant differences compared to the NF-Cas group (* *p* < 0.05), and a number signs indicate significant differences compared to HF-Cas group (^#^ *p* < 0.05). Protein, effect of protein source; Fat, effect of fat content; Days, effect of feeding period; Protein × Fat, interaction between protein source and fat content; Protein × Days, interaction between protein source and feeding period; Fat × Day, interaction between fat content and feeding period; Protein × Fat × Day, interaction between protein source, fat content and feeding period; ns, not significant.

In experiment 1, the Bonferroni multiple comparisons test showed that the gastrocnemius muscle mass of the APP group was significantly higher than that of the Cas group after 2 and 7 days of feeding in rats fed the normal-fat diet (107.5% and 105.2%, respectively), and after 7 days of feeding in rats fed the high-fat diet (105.8%) (Figure 2B). After 2 days of feeding in rats fed the high-fat diet, the gastrocnemius muscle mass was slightly higher in the APP group than that of the Cas group (103.3%); it did not reach the level of significant difference (Figure 2B). However, the ANOVA showed a significant main effect of the protein source on the gastrocnemius muscle mass, but not a significant interaction between protein source and fat content, suggesting that dietary APP increased the gastrocnemius muscle mass, regardless of the dietary fat content (Figure 2B). The EDL muscle mass in the APP group was significantly higher than that in the Cas group after 7 days of feeding in rats fed the high-fat diet (105.1%) but not under other conditions (Figure 2C). However, the three-way ANOVA of EDL muscle mass showed that there was a significant interaction between protein source and feeding period, although this was not the case when fat content was considered (Figure 2C). Similarly, the mass of the gastrocnemius muscle in the APP group was significantly higher than that in the Cas group (110.0%, 109.7%) after 7 and 56 days of feeding in rats fed the normal-fat diet, whereas there were no significant differences in the soleus and EDL muscle mass between the two groups (Figure 2F).

### 3.3. Effects of APP on Muscle Fiber CSA and Type, and Connective Tissue

**Figure 3:**
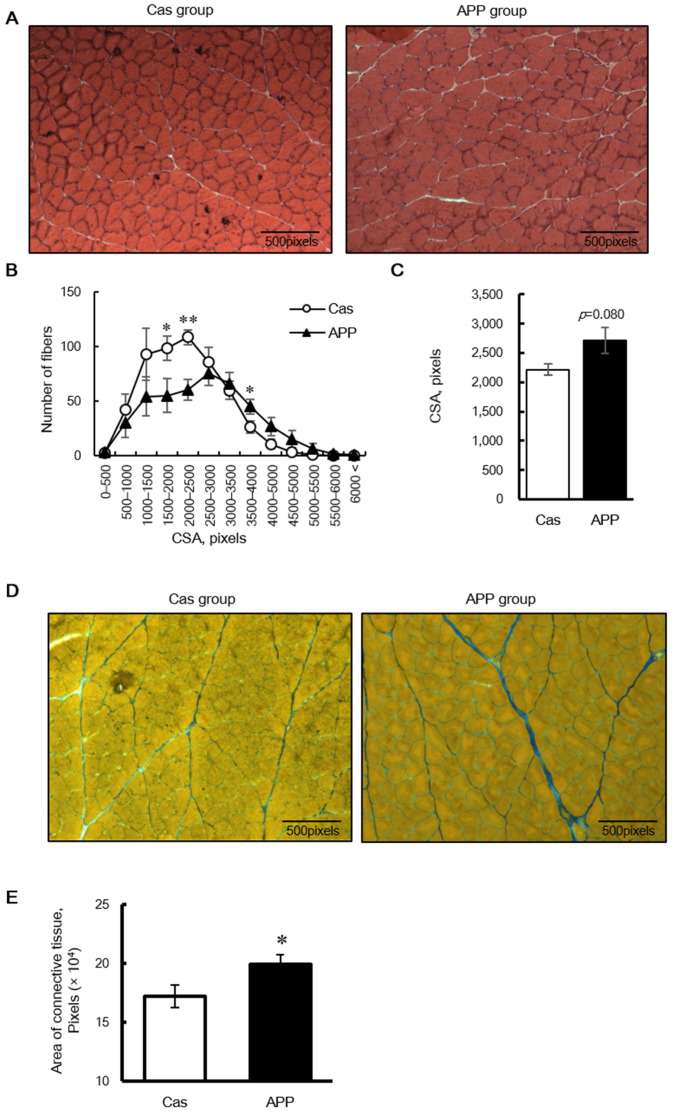
The cross-sectional area (CSA) in muscle fibers and connective tissue in the gastrocnemius muscle of rats fed a normal-fat casein diet or a normal-fat Alaska pollack protein (APP) diet after 7 days of feeding. Transverse sections stained with hematoxylin-eosin (**A**), distribution of CSA (**B**), and mean fiber CSA (**C**) in the gastrocnemius muscle of normal-fat casein diet group (Cas, *n* = 7) and normal-fat APP diet group (APP, *n* = 7) and transverse sections stained with Azan (**D**) and the average area of connective tissue (**E**) in the gastrocnemius muscle of normal-fat casein diet group (Cas, *n* = 8) and normal-fat APP diet group (APP, *n* = 8) are shown. Data are expressed as the mean ± standard error (SEM). Asterisks indicate significant differences relative to the Cas group, as determined using Student’s *t*-test. (*, *p* < 0.05; **, *p* < 0.01).

**Figure 4:**
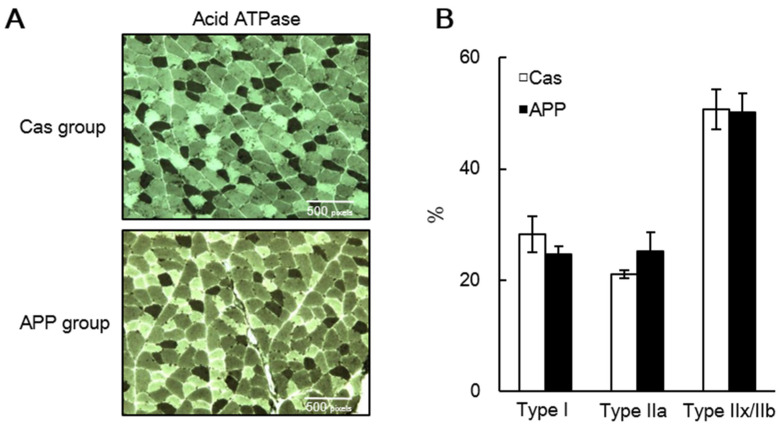
Muscle fiber composition in the gastrocnemius of rats fed a normal-fat casein diet or a normal-fat Alaska pollack protein (APP) diet after 7 days of feeding. Representative transverse sections stained with myosin ATPase preincubated at pH 4.7 (**A**) and percentages of the fiber types (**B**) in the gastrocnemius muscle of normal-fat casein diet group (Cas, *n* = 3) and normal-fat APP diet group (APP, *n* = 4) after 7 days of feeding are shown. Dark and light fibers are types I and II, respectively. Data are expressed as the mean ± standard error (SEM). Statistical analysis was performed using Student’s unpaired *t*-test.

HE staining revealed that the CSA of the fibers shifted toward a larger size in the APP group than that in the Cas group (Figure 1A,B); the mean CSA of the gastrocnemius muscle tended to be higher in the APP group than that in the Cas group (122.5%) (Figure 3C). Azan staining revealed that dietary APP significantly increased the collagen-rich connective tissue (115.7%) (Figure 3D,E). In the gastrocnemius muscle sections stained with myosin ATPase (preincubation pH 4.7), no differences were observed between the Cas and APP groups with respect to the percentage of dark fibers of Type I, lightly stained Type IIA, and intermediate fibers of Type IIX or IIB fibers (Figure 4A,B).

### 3.4. Effects of APP on Gene Expression of Muscle Mass and Metabolism Regulators

**Figure 5:**
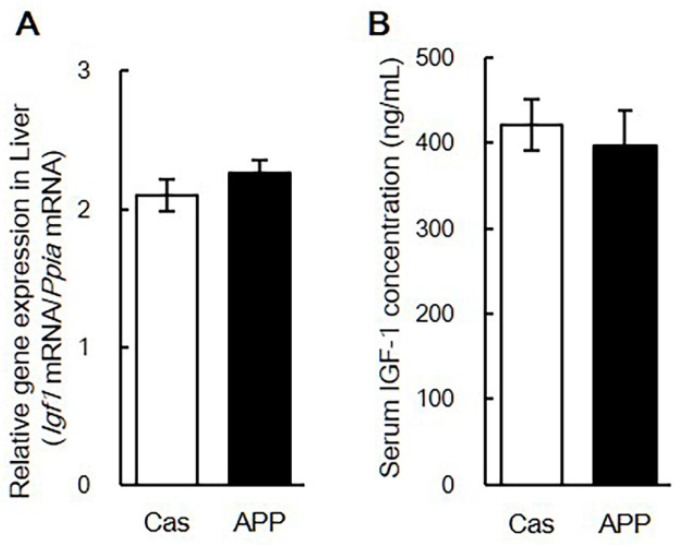
The gene expression of *Igf1* in liver and serum concentration of IGF-1 of rats fed a normal-fat casein diet or a normal-fat Alaska pollack protein (APP) diet after 7 days of feeding. The gene expression of *Igf1* in liver (**A**) and serum concentration of IGF-1 (**B**) in normal-fat casein diet group (Cas, *n* = 12) and normal-fat APP diet group (APP, *n* = 12) after 7 days of feeding are shown. Data are expressed as the mean ± standard error (SEM). Statistical analysis was performed using Student’s unpaired *t*-test.

Quantitative PCR analysis revealed changes in gene expression in the gastrocnemius muscle after 2 days of feeding in rats fed a normal-fat diet. APP intake for 2 days significantly decreased *Fbxo32* and *Trim63* gene expression compared with Cas intake in rats fed the normal diet (55.0%, 75.0%) (Table 4). APP intake for 7 days significantly decreased *Mstn* and *Trim63* gene expression compared with Cas intake in rats fed the normal diet (62.8%, 80.1%) (Table 5). There were no significant differences in the gene expression of MHCs, MRFs, *Igf1*, and *Slc2a4* between the Cas and APP groups after 7 days of feeding (Table 5). There was no significant difference in the gene expression of *Igf1* in the liver and serum IGF-1 concentrations between the Cas and APP groups after 7 days of feeding (Figure 5A,B). There were no significant differences in the expression of MHCs, MRFs, muscle-specific ubiquitin ligases, *Igf1*, *Mstn*, and *Slc2a4* in the gastrocnemius muscle between the Cas and APP groups after 56 days of feeding (Table 6).

### 3.5. Effects of APP on Protein Synthesis

**Figure 6:**
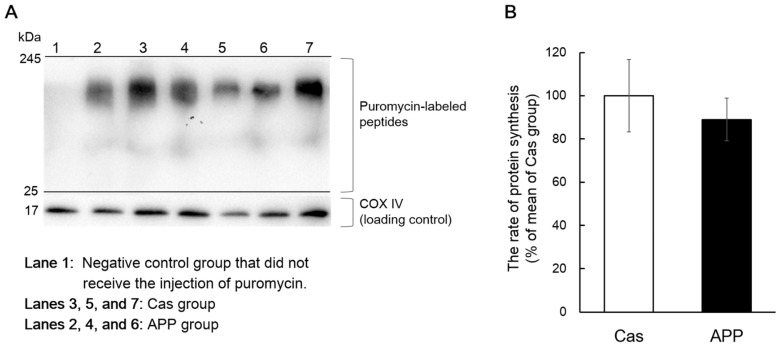
The rates of protein synthesis in the gastrocnemius muscle of rats fed a normal-fat casein diet or a normal-fat Alaska pollack protein (APP) diet after 2 days of feeding. Representative images of Western blot analysis for puromycin-labeled peptides are shown (COX IV is shown as a loading control) (**A**). The rate of protein synthesis in the gastrocnemius muscle of normal-fat casein diet group (Cas, *n* = 5) and normal-fat APP diet group (APP, *n* = 5) after 2 days of feeding are shown (**B**). Data are expressed as the mean ± standard error (SEM). Statistical analysis was performed using Student’s unpaired *t*-test.

Protein synthesis was measured in rats fed the normal-fat diet using the SUnSET method, which revealed changes in the rate of protein synthesis, as reflected by the amount of puromycin incorporated into newly synthesized proteins. There was no significant difference in the protein synthesis, based on the amount of puromycin-labeled peptides, between the Cas and APP groups (Figure 6A,B).

## 4. Discussion

In this study, we demonstrated that dietary APP increased gastrocnemius skeletal muscle mass after only 2 days of feeding, regardless of the dietary fat content (Figure 2B). Furthermore, we showed that the mass of the gastrocnemius muscle in the APP group was significantly higher than that in the Cas group (109.7%) after 56 days of feeding in rats fed the normal-fat diet in experiment 2 (Figure 2F); we have previously shown a similar effect in rats fed the high-fat diets after 56 days (109.8% increase) [11]. Taken together, these results suggest that APP induces acute and sustainable skeletal muscle hypertrophy in rats, regardless of the dietary fat content. In addition, dietary APP increased gastrocnemius skeletal muscle CSA after 7 days of feeding in rats fed the normal diet (Figure 3A–C). These results suggest that dietary APP induces acute and sustainable skeletal muscle hypertrophy, regardless of the dietary fat content. Dietary APP also increased gastrocnemius skeletal muscle collagen-rich connective tissue after 7 days of feeding in rats fed the normal diet (Figure 3D,E). Miller et al. reported that the rates of muscle myofibrillar protein and collagen synthesis increased after a bout of strenuous and non-damaging exercise, suggesting that there is a close relationship between muscle fiber hypertrophy and the development of intramuscular connective tissue components [36]. It is speculated that dietary APP may develop intramuscular connective tissue in a coordinated manner with muscle fiber hypertrophy; however, further studies are needed to determine this.

In fish meat, major proteins are those such as myosin, actin, troponin, tropomyosin and collagen [37]. It is speculated that the peptide derived by digestion of these proteins of APP may promote muscle fiber hypertrophy in a coordinated manner with the development of intramuscular connective tissue. APP and the peptide derived by the digestion of APP reported to have beneficial effects on glucose metabolism [38], the accumulation of visceral fats [39] and insulin intestinal mucosal immunity function [40]. Zdzieblik et al. reported that an oral administration of collagen peptide has a muscle hypertrophy effect with, but not without an intervention of the exercise in the clinical study [41]. However, APP induced muscle hypertrophy without the forced exercise in this study. Therefore, collagen peptide derived by digestion of APP may not be responsible for the hypertrophic effects of APP on the skeletal muscle. It is possible that there are the active peptides derived by digestion of other major proteins of APP for muscle hypertrophy effect, although very little is known about these peptides. Further studies will show which of the peptide derived by digestion of APP is effective in promoting muscle hypertrophy.

The balance of protein synthesis and degradation is controlled primarily by signals originating from IGF-1 [18]. Muscle regeneration and hypertrophy are modulated by the mitotic and myogenic activity of locally produced IGF-1, which functions in an autocrine/paracrine mode. We have previously reported that APP increased the expression of *Igf1* genes in the immobilized limbs after the recovery period in the soleus muscle [14]. In this study, increased gene expression of *Igf1* was not observed after 2, 7, and 56 days of feeding in rats fed a normal diet (Table 4, Table 5 and Table 6) in the gastrocnemius muscle. Furthermore, there were no significant differences in the gene expression of *Igf1* in the liver and serum IGF-1 concentration between the Cas and APP groups (Figure 5). These results suggest that dietary APP may induce muscle hypertrophy independent of an increase in IGF-1 levels. Moreover, dietary APP significantly decreased the gene expression of *Mstn* after 7 days of feeding (Table 5). Myostatin/*Mstn* is a negative regulator of anabolic pathways that inhibit AKT activation in skeletal muscle [18]. This suggests that dietary APP may promote protein synthesis and suppress protein degradation in the skeletal muscle.

It is important to determine the effect of dietary APP on the rate of protein synthesis; therefore, we addressed this question using the SUnSET technique. This method involves incubation with puromycin, an antibiotic that is a structural analog of tyrosyl-tRNA, which can be incorporated into a translating polypeptide chain. The diurnal pattern of muscle protein synthesis peaks during the dark phase, corresponding to a period of increased food intake, increased circulating amino acids, and increased insulin in nocturnal rodents [42]. Food consumption is concentrated at the beginning of the dark phase in the natural food-intake patterns of rodents fed ad libitum [43]. Thus, we expected that dietary APP could increase skeletal muscle protein synthesis at the initial stage of the dark phase (2 h after the start of the dark phase). However, we did not observe an increase in the rate of protein synthesis following APP intake (Figure 6). Our results suggest that dietary APP does not induce acute protein synthesis in the gastrocnemius muscle during the initial dark phase. Skeletal muscle hypertrophy caused by APP intake occurs over a few days. Evaluation over such periods may be needed for assessing the effect of dietary APP on muscle protein synthesis. As one approach, MacDonald et al. reported that muscle protein synthesis could be measured using gas chromatography-pyrolysis-isotope ratio mass spectrometry over a period of 4–14 days following a small single bolus of heavy water in humans [44]. Further studies are needed to determine whether dietary APP promotes muscle protein synthesis over a few days.

Nevertheless, this study revealed that dietary APP significantly decreased the gene expression of MuRF1/*Trim63* after 2 and 7 days of feeding (Table 4 and Table 5) and decreased the gene expression of atrogin-1/*Fbxo32* after 2 days of feeding (Table 4). MuRF1/*Trim63* and atrogin-1/*Fbxo32* are muscle-specific ubiquitin ligases. Their expressions are increased transcriptionally under various atrophy conditions [45,46,47], and mice deficient in either MuRF1/*Trim63* or atrogin-1/*Fbxo32* were observed to be resistant to atrophy [45]. It is speculated that muscle hypertrophy induced by dietary APP may be attributed to the suppression of protein degradation by the ubiquitin–proteasome pathway. Serum 3-methylhistidine appears to be a potential biomarker for elevated muscle protein turnover because it cannot be further metabolized nor can it be reutilized for muscle protein synthesis [48,49]. We measured the serum concentration of 3-methylhistidine in this study, which was significantly higher in the APP group than that in the Cas group (data not shown). The compound 3-methylhistidine is formed in the muscle by the post-translational methylation of histidine residues in actin and myosin [50,51,52]; hence, APP-derived 3-methylhistidine can influence the plasma concentrations of 3-methylhistidine. For measuring endogenously-released 3-methylhistidine in the plasma, it is recommended to adhere to a meat-free diet for at least 3 days before blood sampling [53]. Therefore, it is difficult to assess the effect of diet on skeletal muscle protein degradation. Further studies are needed to determine whether dietary APP may be involved in the suppression of protein degradation through the ubiquitin–proteasome pathway.

In rats fed a high-fat diet, dietary APP significantly decreased the mRNA expression of *Myh7*, a slow-twitch muscle marker, in the soleus muscle, and significantly increased the mRNA expression of *Myh4*, a marker for fast-twitch muscle, in the EDL muscle after 8 weeks of feeding [11]. In addition, the gastrocnemius muscles were whitened by APP intake [11]. In contrast, in rats fed a normal-fat diet, dietary APP increased the muscle fiber diameter in both slow and fast muscle fiber types after 7 days of feeding [54]. In this study, we did not observe increases in the gene expressions of fast-twitch muscle-type markers (fast MHCs and a glucose transporter) in rats fed the normal-fat diet, regardless of the duration of APP intake (Table 4, Table 5 and Table 6). Furthermore, we did not observe any changes in muscle fiber type composition with APP feeding in rats fed the normal-fat diet through myosin ATPase staining (Figure 4). Although, in our previous study, we speculated that APP intake increases fast-type muscle mass and that it may cause a slow-to-fast shift in muscle fiber type [11], we did not observe a relationship between muscle hypertrophy and a slow-to-fast shift in muscle fiber type in this study. Further studies are needed to determine whether high-fat diet feeding is essential for inducing a slow-to-fast shift in muscle fiber type through APP intake.

## 5. Conclusions

We demonstrated that dietary APP induces acute and sustainable skeletal muscle hypertrophy, regardless of dietary fat content. Although we did not observe an increase in the rate of protein synthesis following APP intake, we found that dietary APP causes suppression of the gene expression of negative muscle mass regulators, such as *Mstn*, *Trim63* and *Fbxo32*. It is speculated that muscle hypertrophy induced by dietary APP may be attributed to the suppression of protein degradation by the ubiquitin–proteasome pathway.

## Figures and Tables

**Table 1:** The nutritional profile and amino acid composition of protein sources.

	Cas	APP
Protein, g/100 g	86.2	96.9
Fat, g/100 g	1.0	<0.1
Amino acids, %		
Leucine	8.4	7.5
Valine	5.8	4.5
Isoleucine	4.5	4.0
Methionine	2.5	2.9
Threonine	3.9	4.2
Histidine	2.7	2.2
Phenylalanine	4.6	3.6
Tryptophan	1.1	1.1
Lysine	7.1	8.6
Arginine	3.3	7.0
Glycine	1.7	4.2
Glutamic acid	20.0	14.1
Alanine	2.8	5.5
Tyrosine	5.0	3.3
Aspartic acid	6.4	9.6
Serine	5.2	4.0
Cystine	0.3	1.1
Proline	9.6	3.2

Cas, casein; APP, Alaska pollack protein.

**Table 2:** Compositions of the experimental diets (g/kg diet).

	High-Fat Diet		Normal Diet	
	Cas	APP	Cas	APP
Ingredient, g/kg diet				
Casein	200	-	200	-
APP	-	178	-	178
L-Cysteine	3	3	3	3
α-Cornstarch	257	279	532	554
Sucrose	200	200	100	100
Cellulose	50	50	50	50
Soybean oil	30	30	70	70
Lard	215	215	-	-
AIN-93 mineral mixture	35	35	35	35
AIN-93 vitamin mixture	10	10	10	10
Component, unit/kg diet				
Energy, kcal	4751	4821	3876	3946
Protein, g	172	172	172	172
Fat, g	247	245	72	70
Calcium, mg	5000.0	5135.8	5000.0	5135.8
Phosphorous, mg	3000.0	3054.4	3000.0	3054.4

AIN-93 vitamin mixture contains 2.5 g of choline bitartrate, 30 mg of nicotinic acid, 15 mg of pantothenate, 6 mg of pyridoxine, 5 mg of thiamin, 6 mg of riboflavin, 2 mg of folic acid, 750 μg of vitamin K, 200 μg of D-Biotin, 25 μg of vitamin B-12, 4000 IU of vitamin A, 1000 IU of vitamin D3 and 75I U of vitamin E, 30 U of nicotinic acid/10 g mixture. AIN-93 mineral mixture contains 5000.0 mg of calcium, 1561.0 mg of phosphorus, 3600.0 mg of potassium, 300.0 mg of sulfur, 1019.0 mg of sodium, 1571.0 mg of chloride, 507.0 mg of magnesium, 35.0 mg of iron, 30.0 mg of zine, 10.0 mg of manganese, 6.0 mg of copper, 0.2 mg of iodine, 0.15 mg of molybdenum, 0.15 mg of selenium, 5.0 mg of silicon, 1.0 mg of chromium, 1.0 mg of fluoride, 0.5 mg of nickel, 0.5 mg of boron, 0.1 mg of lithium, and 0.1 mg of vanadium/35 g mixture [12]. Cas, casein; APP, Alaska pollack protein.

**Table 3:** Primer sequences.

Genes	Sequences		Product Size (bp)
	Sense	Antisense	
*Myh7*	TTCATTGATAGCCGGAAAGG	TTGGTGTGGCCAAACTTGTA	83
*Myh2*	CCCTCCCAAGTACGACAAGA	CACAGAAGAGGCCCGAGTAG	128
*Myh1*	CAGCTTACCGAGGCAAAAAG	TCCCGATCTGTCAACATGAA	91
*Myh4*	ATAGCTCGCAGCCATGAGTT	CTCGATTCGCTCCTTTTCAG	91
*Myf5*	CCACCTCCAACTGCTCTGAT	CAGGGCAGTAGATGCTGTCA	90
*Myod1*	GGGGTTCAGGAGTGACAGAA	CGGCGATAGTAGCTCCATGT	95
*Myog*	CCTGCCCTGAGATGAGAGAG	TGGAAGGTTCCCAATATCCA	91
*Myf6*	TGTACCCAGGGAGTGATGGT	AACGTGTTCCTCTCCACTGC	89
*Igf1*	CTTGAGCAACCTGCAAAACA	GGAAATGCCCATCTCTGAAA	80
*Mstn*	GATGGGCTGAATCCCTTTTT	CCGTGGAGTGTTCATCACAG	94
*Fbxo32*	AAGCTTGTGCGATGTTACCC	CCAGGAGAGAATGTGGCAGT	81
*Trim63*	AGTCGCAGTTTCGAAGCAAT	CTGCTTCTCCAGGTTCTCCA	80
*Slc2a4*	CTGAGTTCAGGGGGATGAGA	AGTTCAGTGAAAGCGCCAGT	89
*Ppia*	ATGGTCAACCCCACCGTGTT	CGTGTGAAGTCACCACCCT	206

**Table 4:** The effect of APP for 2 days on gene expressions of myosin heavy chains and regulators of muscle mass and metabolism in the gastrocnemius muscles in rats fed a normal-fat diet.

		Cas	APP
		(Target Gene mRNA/*Ppia* mRNA)	
*Myh7*	(×10^−2^)	1.57 ± 0.27	0.96 ± 0.14
*Myh2*	(×10^−2^)	2.50 ± 0.31	2.07 ± 0.26
*Myh1*	(×10^−2^)	4.18 ± 0.49	3.59 ± 0.56
*Myh4*	(×10^−1^)	0.74 ± 0.12	0.63 ± 0.06
*Myf5*	(×10^−3^)	3.57 ± 0.37	2.86 ± 0.27
*Myod1*	(×10^−3^)	3.26 ± 0.43	2.81 ± 0.37
*Myog*	(×10^−2^)	4.18 ± 0.40	3.72 ± 0.51
*Myf6*	(×10^−2^)	6.02 ± 0.50	5.99 ± 0.60
*Igf1*	(×10^−1^)	3.89 ± 0.39	3.81 ± 0.36
*Mstn*	(×10^−2^)	2.25 ± 0.24	1.71 ± 0.21
*Fbxo32*	(×10^−3^)	1.89 ± 0.21	1.04 ± 0.11 **
*Trim63*	(×10^−2^)	1.64 ± 0.14	1.23 ± 0.09 *
*Slc2a4*	(×10^−1^)	1.77 ± 0.21	1.21 ± 0.05

The gene expressions of myosin heavy chains and regulators of muscle mass and metabolism of normal fat-casein diet group (Cas, *n* = 8) and normal fat-APP diet group (APP, *n* = 8) after 2 days of feeding are shown. Data are expressed as the mean ± standard error (SEM). Asterisks indicate significant differences compared to the Cas group by unpaired Student’s *t*-test (*, *p* < 0.05; **, *p* < 0.01).

**Table 5:** Effect of APP for 7 days on gene expressions of myosin heavy chains and regulators of muscle mass and metabolism in the gastrocnemius muscles in rats fed a normal-fat diet.

		Casein	APP
		(Target Gene mRNA/*Ppia* mRNA)	
*Myh7*	(×10^−2^)	1.88 ± 0.31	1.97 ± 0.38
*Myh2*	(×10^−2^)	5.38 ± 0.67	4.88 ± 0.36
*Myh1*	(×10^−2^)	3.65 ± 0.53	3.98 ± 0.47
*Myh4*	(×10^−1^)	4.48 ± 0.27	4.77 ± 0.44
*Myf5*	(×10^−3^)	3.03 ± 0.17	2.86 ± 0.18
*Myod1*	(×10^−3^)	2.04 ± 0.31	1.68 ± 0.05
*Myog*	(×10^−2^)	2.70 ± 0.14	2.95 ± 0.25
*Myf6*	(×10^−2^)	4.49 ± 0.26	3.88 ± 0.40
*Igf1*	(×10^−1^)	2.91 ± 0.10	2.57 ± 0.26
*Mstn*	(×10^−2^)	1.56 ± 0.07	0.98 ± 0.13 **
*Fbxo32*	(×10^−3^)	2.00 ± 0.30	1.61 ± 0.22
*Trim63*	(×10^−2^)	1.51 ± 0.10	1.21 ± 0.08 *
*Slc2a4*	(×10^−1^)	9.90 ± 0.76	10.35 ± 0.95

The gene expressions of myosin heavy chains and regulators of muscle mass and metabolism of normal fat-casein diet group (Cas, *n* = 8) and normal fat-APP diet group (APP, *n* = 8) after 7 days of feeding are shown. Data are expressed as the mean ± standard error (SEM). Asterisks indicate significant differences compared to the Cas group by unpaired Student’s *t*-test (*, *p* < 0.05; **, *p* < 0.01).

**Table 6:** Effect of APP for 56 days on gene expressions of myosin heavy chains and regulators of muscle mass and metabolism in the gastrocnemius muscles in rats fed a normal-fat diet.

		Casein	APP
		(Target Gene mRNA/*Ppia* mRNA)	
*Myh7*	(×10^−1^)	1.05 ± 0.18	1.29 ± 0.12
*Myh2*	(×10^−2^)	8.01 ± 0.51	8.63 ± 0.74
*Myh1*	(×10^−1^)	9.11 ± 0.83	9.84 ± 0.90
*Myh4*	(×10^−1^)	1.16 ± 0.08	1.12 ± 0.07
*Myod1*	(×10^−3^)	5.09 ± 0.59	5.80 ± 0.54
*Myog*	(×10^−2^)	4.53 ± 0.47	5.28 ± 0.38
*Myf6*	(×10^−1^)	1.89 ± 0.11	1.65 ± 0.06
*Igf1*	(×10^−1^)	6.75 ± 0.37	7.43 ± 0.34
*Mstn*	(×10^−2^)	1.39 ± 0.16	1.26 ± 0.09
*Fbxo32*	(×10^−3^)	1.09 ± 0.09	0.92 ± 0.10
*Trim63*	(×10^−2^)	4.96 ± 0.34	5.24 ± 0.48
*Slc2a4*	(×10^−1^)	5.12 ± 0.32	4.99 ±0.21

The gene expressions of myosin heavy chains and regulators of muscle mass and metabolism of normal fat-casein diet group (Cas, *n* = 10) and normal fat-APP diet group (APP, *n* = 10) after 56 days of feeding are shown. Data are expressed as the mean ± standard error (SEM). Statistical analysis was performed using the Student’s unpaired *t*-test.

## Data Availability

The data that support the findings of this study are available from the corresponding author upon reasonable request.

## Supplementary Materials

The following are available online at https://www.mdpi.com/article/10.3390/nu14030547/s1, Table S1: The average, standard deviation (SD) and Coefficients of variation (CqCV%, SD/mean·100) of Cq for *Ppia* gene for each of the different groups.

## Abbreviations

AKT	protein kinase B
APP	Alaska pollack protein
atrogin-1	atrophy gene-1
Cas	casein
CSA	cross-sectional area
EDL	extensor digitorum longus
HE	Hematoxylin and eosin
IGF-1	insulin-like growth factor 1
MHC	myosin heavy chain
MRF	myogenic regulatory factor
MTOR	mechanistic target of rapamycin
MuRF1	muscle-specific RING finger protein-1
Cq	quantification cycle
SUnSET	surface sensing of translation
TBS	Tris-buffered saline
TBST	Tris-buffered saline with 0.1% Tween 20
