# Supplementary material for: Dietary Alaska Pollack Protein Induces Acute and Sustainable Skeletal Muscle Hypertrophy in Rats
Source: Nutrients. 2022 Jan 27;14(3):547. doi: 10.3390/nu14030547 (PMC8837972; doi:10.3390/nu14030547)
Supplement: Supplementary file 1 [file nutrients-14-00547-s001.zip › nutrients-1525546-supplementary.pdf]

Uchida K *et al*. Dietary Alaska pollack protein induces acute and sustainable skeletal muscle hypertrophy in rats

Supplementary table

Table S1. The average, standard deviation (SD) and Coefficients of variation (CqCV%, SD/mean·100) of Cq for *Ppia* gene for each of the different groups.

	2-days feeding		7-days feeding		56-days feeding	
	Cas	APP	Cas	APP	Cas	APP
Average	18.17	17.93	18.31	18.16	21.19	20.98
SD	0.47	0.31	0.19	0.19	0.48	0.42
CqCV%	2.56	1.74	1.06	1.05	2.25	1.99

Cas, casein; APP, Alaska pollack protein.
